# Ethical and definitional considerations in research on child sexual violence in India

**DOI:** 10.1186/s12889-018-6036-y

**Published:** 2018-09-27

**Authors:** Radhika Dayal, Ameeta S. Kalokhe, Vikas Choudhry, Divya Pillai, Klaus Beier, Vikram Patel

**Affiliations:** 1 Public Health Foundation of India, Plot No. 47, Sector 44, Institutional Area, Gurugram, 122002 Haryana India; 2 Emory University School of Medicine Division of Infectious Diseases and Rollins School of Public Health Department of Global Health, Atlanta, USA; 3 Sambodhi Research and Communications Pvt. Ltd., C-126, Sector- 2, Noida, 201301 Uttar Pradesh India; 4 Institute of Sexology and Sexual Medicine, Charité - Universitätsmedizin Berlin, corporate member of Freie Universität Berlin, Humboldt-Universität zu Berlin, and Berlin Institute of Health, Luisenstraße 57, 10117 Berlin, Germany; 5 Department of Global Health and Social Medicine, Harvard Medical School, MA Boston, 02115 USA

**Keywords:** Ethical guidelines, Child sexual abuse, Child sexual violence, India

## Abstract

**Background:**

While critically important, child sexual violence (CSV) research poses numerous ethical and safety challenges. Recently, the studies dedicated to understanding and addressing CSV in India have been on the rise, but no published ethical guidelines to direct such research currently exist. To help inform ethical and safety recommendations for the design, conduct, and reporting of future CSV research in India and similar settings, we systematically reviewed the ethics and safety practices reported in recent Indian CSV literature.

**Methods:**

A multi-tiered approach was used to understand current ethical practices and gaps: 1) systematic review of Indian CSV studies published over the past decade, 2) examination of existing guidelines on related topics to develop an ethical framework, 3) development of an ethics checklist based on the recommendations from the surveyed guidelines, and 4) application of the checklist to each of the reviewed studies.

**Result:**

Our search yielded 51 eligible studies. From each, data from 6 major thematic areas was extracted: informed consent, confidentiality, selection, training, and protection of study team members, validity of CSV measurement methods, measures to minimize participant harm, and participant compensation. Several gaps were noted: only two-thirds reported approval by ethics committees, obtaining informed consent, and assured participants of confidentiality. Only 25% (13/51) reported assessing ongoing CSV risk and providing necessary support services, none noted whether ongoing CSV was reported to authorities (required by Indian law), and none reported safeguards to protect staff from the effects of conducting CSV research. Further, 43% (22/51) limited surveillance of CSV to one form of abuse and/or used a “loaded term,” increasing the potential for underreporting.

**Conclusions:**

Through enhancing understanding of current ethical practices and gaps in CSV research in India, this systematic review informs reporting protocols and future guidelines for CSV research in India and other similar settings.

## Background

The World Health Organization defines child sexual violence (CSV) as “the involvement of a child in sexual activity that he or she does not fully comprehend, is unable to give informed consent to, or for which the child is not developmentally prepared and cannot give consent, or that violates the laws or social taboos of society.” [1] In 2007, the India Ministry of Women and Child Development (MWCD) conducted the most extensive national survey of child abuse in India to date [2]. While it employed a purposive rather than random sample, (drawing children from 5 select groups: street children, working children, children in institutional care, children in school, and children in families not attending schools, from 13 of 28 states), it began to shed light on the magnitude and extent of CSV in India. Over half (53%) of the 12,447 respondents, reported experience of one or more forms of CSV, and notably, 53% of those reporting abuse were boys and 47% girls [2].

In addition to being prevalent, CSV violates basic human rights, and is associated with poor mental health (i.e. depression, anxiety, panic disorders, substance abuse disorders, and attempted suicide), poor physical health (i.e. injury, sexually transmitted infections), unintended pregnancy, social harm (i.e. difficulty sustaining relationships, missed school), and increased risk of intimate partner violence later in life [3]. The high prevalence and associated morbidity speak to the need for effective evidence-based prevention and management strategies. While critically important, CSV research poses numerous ethical and safety challenges. A recent review conducted by UNICEF [4] highlights four key ethical dilemmas and questions in the field: 1) the negative potential impact of the research on the child, 2) the extent of study information to provide to the parents and child, 3) the capacity for a child to provide informed consent, and 4) the need to weigh maintenance of confidentiality versus child protection.

While many aspects of these dilemmas are universal, they also need to be examined in the socio-cultural context of India. For example, the strong cultural taboos associated with discussing sex and sexual violence in India may result in greater emotional distress for the participant of a CSV study, or alternatively, a stronger sense of relief from the opportunity to openly discuss one’s experience. Further, Indian parents traditionally tend to be heavily involved in their children’s decision-making, so children may feel uncomfortable independently making decisions surrounding informed consent. Mandatory reporting laws invoked by the India 2012 Protection of Children from Sexual Offenses Act (POCSO) to enhance child protection may result in inadvertent harm (i.e. perpetrator retribution, heightened survivor stigma, labeling, and blaming) due to exposure of abuse history in the setting of saturated enforcement, legal, and community support systems [4–6]. Lastly, use of computer-based data collection tools to help maintain confidentiality and potentially work around the mandatory reporting laws may be limited by the availability of required technology and associated literacy.

Clearly, evidence-based, culturally-tailored guidelines that suggest strategies for addressing these ethical dilemmas are needed to guide future CSV research in India and similar research settings abroad. To date, there are no universal, regional, or India-specific guidelines for the conduct of CSV research. To better understand present ethical and safety standards followed in India, we herein systematically review the past decade of Indian CSV literature and examine the ethics and safety practices they reported. As the field of CSV research and evidence-based intervention design in India is young but gaining momentum, the knowledge imparted from this review could help inform ethical and safety recommendations for the design, conduct, and reporting of CSV research in India in the years to come.

## Methods

This review utilized a multi-tiered approach: 1) conduct of a systematic review of the past-decade of CSV studies conducted in India, 2) review of existing guidelines that could help inform an ethical framework for the future conduct of CSV research, 3) development of an ethics checklist based on the recommendations from the surveyed guidelines, and 4) application of the ethics checklist to each of the studies found in our CSV systematic review.

### The systematic review

This study draws from a broader systematic review which aimed to examine the prevalence, determinants and consequences of CSV in India *(under review)*. The review explored articles published in peer-reviewed journals, indexed in PubMed, POPLINE, and PsycINFO, that described CSV experience or perpetration in India. To be included in the review, the articles had to meet the following criteria: i) be written in English, ii) be published between January 1, 2006 and January 1, 2016, iii) involve human subjects, and iv) collect original data on experience, perpetration or response to CSV in India. Searches were conducted using 55 search terms, each paired with the term, “India” (see Table 1).

**Table 1:** SEARCH TERMS PAIRED WITH “INDIA”

Sexual violence	Rape	Sexual Violence Survivor	Adult survivors of child adverse event	Gender based violence
Sexual abuse	Prostitution	Child abuse survivor	Psychological sexual dysfunction	Human trafficking
Sexual assault	Crime victims	Sexual offender	Female genital mutilation	Intimate partner violence
Sexual coercion	Incest	Paedophilia	Domestic violence	Emotional violence
Sexual aggression	Perpetrator	Sexual abuse dysfunction	Sexual maltreatment	Sexually harmful behaviour
Sexual offense	Paedophile	Pornography	Paraphilic disorder	Sexual exploitation
Sexual victim	Sodomy	Non consensual sex	Cyber sexual crime	Sexual harassment
Transvestism	Dating violence	Marital rape	Sexual deviance	Atypical sexual behaviour
Exhibitionism	Physical violence	Abusive images	Juvenile delinquency	Battered child syndrome
Voyeurism	Masochism	Molestation	Exposure to violence	Effects of violence
Fetishism	Stalking	Sexual crime	Hebephilia	Online sexual offender

**Fig. 1:**
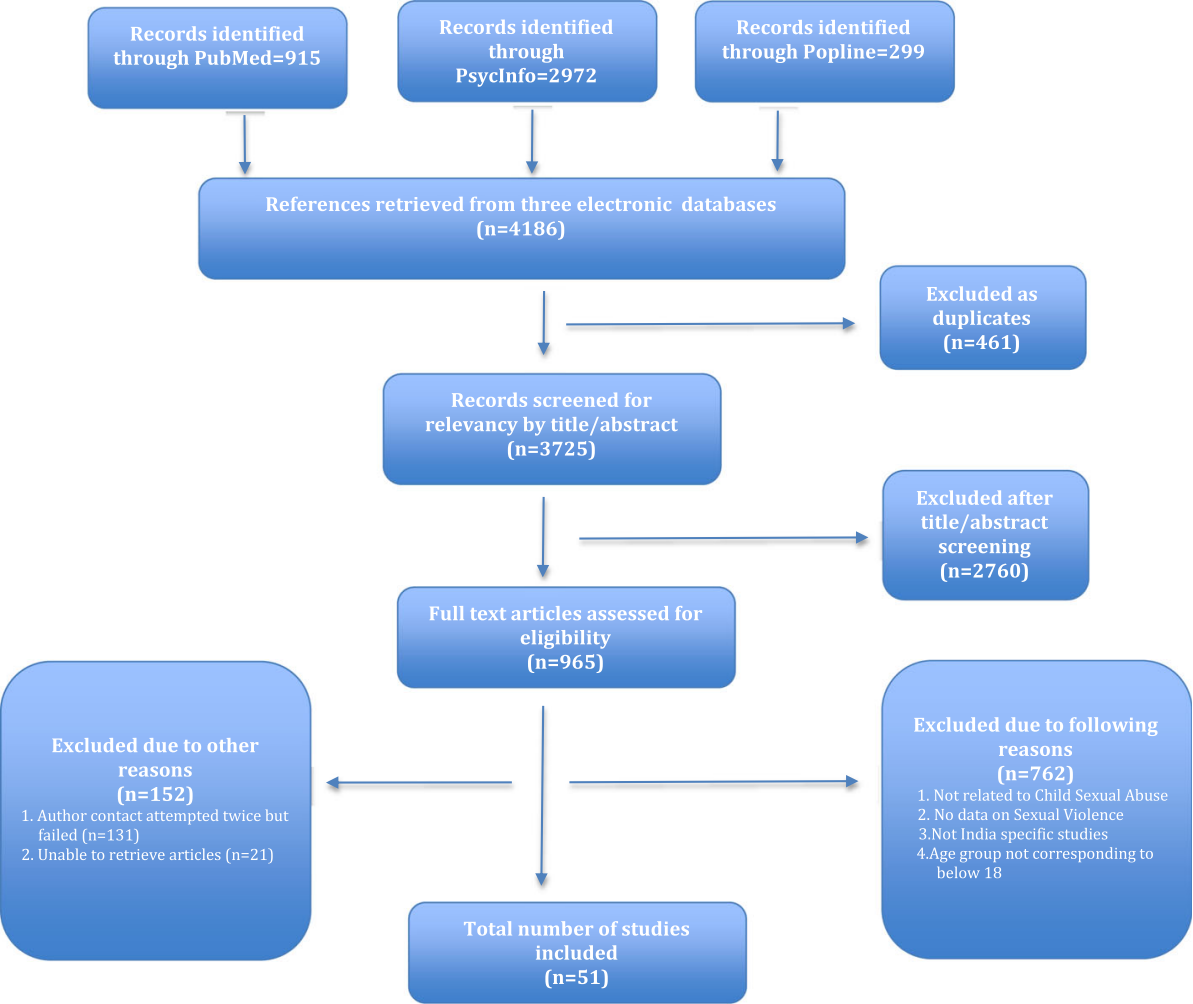
Adapted PRISMA Flow Chart demonstrating study selection and filter results [29]

The initial search yielded 4186 articles. After removal of duplicates, 3725 were left and screened for potential relevance by title and abstract review. Through title and abstract screening, 2760 studies were excluded based on irrelevancy, leaving 965 for full-text screening. Of the 965 articles, 762 articles were excluded because they did not meet eligibility criteria, 131 were excluded as insufficient information was provided to assess eligibility (with two failed attempts to contact authors for additional information), and 21 were excluded because the full text was not found through our institutional libraries nor through repeated attempts to contact authors. The final list of included articles consists of 51 studies. Figure 1 depicts the PRISMA Flow Diagram [29].

### Development of the consolidated ethics checklist

To inform the development of the ethics checklist a web-based search was conducted for guidelines, recommendations, and checklists from public health authorities guiding the design, conduct, and/or reporting of research on child abuse, gender-based violence, and sexual violence. Additionally, we sought input from India CSV experts for guidelines of relevance. Both methods led to author consensus that the following six documents were of greatest relevance for developing an ethics framework to evaluate CSV research in India: the UNICEF review “Ethical Principles, dilemmas, and risks in collecting data on violence against children,” the WHO “Putting Women First: Ethical and Safety Recommendations for Research on Domestic Violence Against Women,” the Sexual Violence Research Initiative “Ethical and Safety Recommendations for Research on the Perpetration of Sexual Violence,” the UNICEF “Guidelines on the Protection of Child Victims of Trafficking”, the Indian Council of Medical Research (ICMR) “National Ethical Guidelines for Biomedical Research Involving Children,” and the WHO clinical guidelines “Responding to Children and Adolescents Who Have Been Sexually Abused.” [4, 7–11]. To develop the Ethics Checklist, key themes from each of the four documents were first extracted and commonalties between documents noted. The key themes were then discussed among the entire research team for inclusion based on their relevance to CSV research until consensus for inclusion was reached. The Ethics Checklist is presented in Table 2. The checklist takes into consideration the following key ethical principles: i) *informed consent, ii) confidentiality, iii) selection, training, and protection of study team members, iv) validity of CSV measurement methods, v) measures to minimize participant harm and vi) participant compensation.* Each of the 51 articles were reviewed by a single author (RD) and information regarding these key ethical principles was extracted. When questions arose or clarifications were needed, RD consulted a second author (AK) to reach consensus. If the information was not reported in a manuscript, we stated so.

**Table 2:** Consolidated Ethics Criteria

1. Does the manuscript report whether informed consent was obtained from the participant and/or permission was obtained from the parent/guardian (where applicable)?	
2. Does the description of informed consent process state that the participant was informed of the limits of confidentiality (i.e. any data that would need to be reported to authorities if it were disclosed during the study)?	
3. Does the manuscript discuss methods used for ensuring participant confidentiality?	
a. Mention not disclosing the true intent of the study until alone with the participant and/or the participant’s parent/guardian?	
b. Mention measures to ensure data confidentiality?	
c. Take care to not present data that could identify the participant?	
4. Does the manuscript discuss how the study team members were selected and trained?	
5. Does the manuscript discuss procedures in place to protect the safety and health of its study team members (i.e. debriefing meetings, limiting time in interviews, self-completion methods, provision of counseling services?)	
6. Were the methods of screening for CSV robust (i.e. survey a range of behaviors vs. use 1–2 question; avoid “loaded” terms like ‘rape,’ ‘violence,’ and ‘abuse’?)	
7. Does the manuscript describe measures for assessing and minimizing participant harm to the participant?	
a. If applicable, was risk of additional/ongoing CSV assessed? If so, does the manuscript discuss whether and how such situations were reported or handled?	
b. Does the manuscript discuss measures to minimize distress?	
c. Does the manuscript discuss whether participants in distress were offered support resources and/or referrals?	

## Results

Among the 51 studies included in the review (Table 3, 4 and 5), 69% (35/51) of studies utilized quantitative methods, 22% (11/51) utilized qualitative methodologies, and 10% (5/51) utilized mixed-methods. The vast majority of quantitative studies employed a cross-sectional design (32/51) with others using designs like case-control or medical case series. Three-fourth (76% or 39/51) specified obtaining institutional ethics approval from their respective institutions. In this section, we systematically discuss each ethics principle and examine how the studies (*n* = 51) have reported information on each of the ethical and safety practices reported in Table 2.

**Table 3:** Ethics Checklist on Confidentiality

	*N* = 51	*N* = 33/51	*N* = 32/51	*N* = 24/51		
References	Age range (in years)	Informed Consent and/or Permission *(P, G, B, NR, NA)*	Participant notification of limits of confidentiality (Y/NR)	Report methods for enhancing privacy and confidentiality? (Y/NR)		
				Report only discussing true study intent in private	Report measures of enhancing data confidentiality	Ensure not identifying data in paper
Charak 2015 [27]	13–17	P	Y	NR	Y	Y
Jaisoorya et al. (2015) [30]	12–18	B	Y	NR	Y	Y
Sahay et al. (2013) [31]	12–19	B	Y	NR	NR	NR
Miller et al. (2014)# ** [32]	10–16	NR	NR	NR	NR	NR
Hasnain et al. (2016) [33]	> 18	NR	Y	NR	Y	NR
Das et al. 2014** [34]	10–16	B	NR	NR	NR	NR
Zolotor et al. (2009) [17]	12–17	P	Y	NR	Y	Y
Deb et al. (2012) [35]	14–19	P	Y	NR	NR	Y
Deb et al. (2010) [36]	14–19	P	Y	NR	NR	Y
Charak 2014 [26]	13–17	P	Y	NR	N	NR
Krishnakumar et al. (2014) [37]	15–19	NR	Y	NR	Y	NR
Sahay et al. (2013) ** [25]	8–30	P	Y	NR	Y	Y
Bhilwar et al. (2015) [18]	18–25	B	Y	NR	Y	Y
Jaya et al. (2007) [38]	15–17	B	Y	Y	Y	NA
Dunne et al. (2009) [19]	18–26	P	Y	NR	NR	NR
Nayak et al. (2010) [39]	16–49	NR	NR	NR	NR	NR
Sahay et al. (2010)** [40]	10–18	NR	Y	NR	Y	NR
Pillai et al. (2008) [41]	12–16	B	NR	NR	NR	NR
Bhattacharya et al. (2012) [42]	Not specified	NA	NR	NA	NA	NA
Kar et al. 2007 [43]	20–58	P	Y	NR	Y	Y
Silverman et al. (2007) [13]	< 18	NA	NR	NA	NA	NA
Silverman et al. (2006) [44]	9–30	NA	NR	NA	NA	NA
Silverman et al. (2011) [45]	≥18	NR	NR	NR	NR	NR
Deb et al. (2008)** [46]	< 18- > 33	NA	NR	NA	NA	NA
Devine et al. (2010)** [47]	≥18	NR	NR	NR	NR	NR
Shahmanesh et al.(2009)a [21]	> 18	NR	Y	Y	Y	NR
Shahmanesh et al.(2009) b [22]	> 18	NR	Y	Y	Y	NR
Bhat et al. (2012) [48]	11–18	B	Y	NR	NR	NR
Deb et al.(2009) [49]	13–18	P	Y	NR	NR	NR
Deb et al. 2011 a# [50]	13–18	P	Y	NR	NR	NR
Deb et al. 2011 b# [51]	13–18	P	Y	NR	NR	NR
Banerjee et al.(2008) [52]	8–14	NR	NR	NR	NR	NR
Jangam et al. (2015) [53]	18–50	P	Y	NR	NR	NR
Reed et al. (2013) [23]	18–40	P	NR	NR	NR	NR
Silverman et al. (2014) [54]	> 18	P	NR	NR	NR	NR
Gaidhane et al. (2008) [55]	11–19	P	Y	NR	Y	NR
Bal et al. (2010) [12]	11–15	G	Y	NR	Y	Y
Tomori et al. 2016** [56]	≥18 years	P	Y	NR	NR	NR
Sahay et al. 2008 [57]	15–26	NR	Y	NR	Y	NR
Karandikar et al. 2013 [58]	20–60	P	Y	NR	NR	NR
Rashid 2012 [24]	13–18	NR	Y	Y	Y	Y
Basu 2012 [59]	Not specified	NR	NR	NR	NR	NR
Karandikar et al. 2013 [28]	20–33	P	Y	Y	Y	NR
Magar 2013# [14]	12–18	NR	NR	NR	NR	NR
Mimiaga 2015 [60]	> 18	P	NR	NR	NR	NR
Gupta 2009 [16]	14–30	P	NR	NR	NR	NR
Sahoo 2015 [15]	16–24	B	Y	NR	Y	Y
Chakrapani 2008 [61]	21–52	P	NR	NR	NR	NR
Sinha 2015 [62]	22–50	P	Y	NR	Y	NR
Pillai et al. (2011) ^##^[63]	16–18	P	Y	NR	NR	NR
Balaji et al. (2011) ^##^ [64]	16–18	P	NR	NR	NR	NR

*Ref* reference, *P* IC from participant, *G* guardian, *B* both, *NR* not reported, *NA* not applicable, *S* selection, *T* training

# Data based on an intervention study

## Data based on the same Intervention Study. The study was conducted with respondents between 16 and 24 years of age, but the analysis was restricted to respondents between 16 and 18 years of age for the purpose of this review

**Mixed Methods study

**Table 4:** Ethics Checklist on selection and training of study team members

	*N* = 35/51
References	Report methods for study team selection and training
Charka 2015 [27]	S
Jaisoorya et al. (2015) [30]	B
Sahay et al. (2013) [31]	NR
Miller et al. (2014)# ** [32]	NR
Hasnain et al. (2016) [33]	NR
Das et al. 2014** [34]	NR
Zolotor et al. (2009) [17]	B
Deb et al. (2012) [35]	S
Deb et al. (2010) [36]	NR
Charak 2014 [26]	T
Krishnakumar et al. (2014) [37]	S
Sahay et al. (2013) ** [25]	S
Bhilwar et al. (2015) [18]	B
Jaya et al. (2007) [38]	B
Dunne et al. (2009) [19]	T
Nayak et al. (2010) [39]	NR
Sahay et al. (2010)** [40]	NR
Pillai et al. (2008) [41]	T
Bhattacharya et al. (2012) [42]	NR
Kar et al. 2007 [43]	S
Silverman et al. (2007) [13]	NR
Silverman et al. (2006) [44]	NR
Silverman et al. (2011) [45]	S
Deb et al. (2008)** [46]	NA
Devine et al. (2010)** [47]	T
Shahmanesh et al.(2009)a [21]	T
Shahmanesh et al.(2009) b [22]	T
Bhat et al. (2012) [48]	S
Deb et al.(2009) [49]	S
Deb et al. 2011 a# [50]	B
Deb et al. 2011 b# [51]	B
Banerjee et al.(2008) [52]	S
Jangam et al. (2015) [53]	T
Reed et al. (2013) [23]	S
Silverman et al. (2014) [54]	S
Gaidhane et al. (2008) [55]	S
Bal et al. (2010) [12]	NR
Tomori et al. 2016** [56]	S
Sahay et al. 2008 [57]	NR
Karandikar et al. 2013 [58]	S
Rashid 2012 [24]	NR
Basu 2012 [59]	S
Karandikar et al. 2013 [28]	S
Magar 2013# [14]	NR
Mimiaga 2015 [60]	NR
Gupta 2009 [16]	S
Sahoo 2015 [15]	S
Chakrapani 2008 [61]	S
Sinha 2015 [62]	S
Pillai et al. (2011) ^##^ [63]	T
Balaji et al. (2011) ^##^ [64]	T

*S* selection, *B* both, *T* training, *NR* not reported, *NA* not applicable

# Data based on an intervention study

## Data based on the same Intervention Study. The study was conducted with respondents between 16 and 24 years of age, but the analysis was restricted to respondents between 16 and 18 years of age for the purpose of this review

**Mixed Methods study

**Table 5:** Ethics checklist on validity of CSV measurement methods, minimization of participant harm and participant compensation

	*N* = 51	*N* = 51	*N* = 13/51		*N* = 14/51
References	Age range (in years)	CSV Measurement methods	Report measures for minimizing harm *(Y/NR)*		Incentives given to the participants (Y,NR,NA)
			Was risk of ongoing CSV assessed?	Reported methods to minimize distress?	
Charka 2015 [27]	13–17	Childhood Trauma Questionnaire	NR	NR	Y
Jaisoorya et al. (2015) [30]	12–18	4 questions on experience of sexual abuse	NR	Y	NR
Sahay et al. (2013) [31]	12–19	Not Stated	NR	NR	NR
Miller et al. (2014)# ** [32]	10–16	Sexual violence perpetration	NR	NR	NR
Hasnain et al. (2016) [33]	> 18	Biographical inventory developed with CSV questions	NR	NR	NR
Das et al. 2014** [34]	10–16	Not Stated	NR	NR	NR
Zolotor et al. (2009) [17]	12–17	ISPCAN Child Abuse Screening Tool Children’s Version (ICAST-C)	NR	NR	NR
Deb et al. (2012) [35]	14–19	ISPCAN**** Child Abuse Screening Tool Children’s Version (ICAST-C)	NR	NR	NR
Deb et al. (2010) [36]	14–19	ISPCAN**** Child Abuse Screening Tool Children’s Version (ICAST-C)	NR	NR	NR
Charak 2014 [26]	13–17	Childhood Trauma Questionnaire	N	N	Y
Krishnakumar et al. (2014) [37]	15–19	Adapted MoWCD*** questionnaire on child abuse	NR	NR	NR
Sahay et al. (2013) ** [25]	8–30	Not Stated	Y	Y	NR
Bhilwar et al. (2015) [18]	18–25	Adapted MoWCD*** questionnaire on child abuse	NR	NR	NR
Jaya et al. (2007) [38]	15–17	Not Stated	NR	NR	NR
Dunne et al. (2009) [19]	18–26	ISPCAN Child Abuse Screening Tools Retrospective version (ICAST-R)	NR	NR	NR
Nayak et al. (2010) [39]	16–49	2 questions on childhood sexual Victimization	NR	NR	NR
Sahay et al. (2010)** [40]	10–18	Non standardized Questionnaire	NR	NR	NR
Pillai et al. (2008) [41]	12–16	Non standardized Questionnaire	Y	Y	NR
Bhattacharya et al. (2012) [42]	Not specified	Clinical case History	NA	NA	NA
Kar et al. 2007 [43]	20–58	Sexual functioning questionnaire	NR	NR	NR
Silverman et al. (2007) [13]	< 18	Not stated	NA	NA	NA
Silverman et al. (2006) [44]	9–30	Not stated	NA	NA	NA
Silverman et al. (2011) [45]	≥18	Not stated	NR	NR	Y
Deb et al. (2008)** [46]	< 18- > 33	Not stated	NA	NA	NA
Devine et al. (2010)** [47]	≥18	Not stated	NR	NR	NR
Shahmanesh et al.(2009)a [21]	> 18	Not stated	NR	Y	Y
Shahmanesh et al.(2009) b [22]	> 18	Not stated	NR	Y	Y
Bhat et al. (2012) [48]	11–18	Finkelhor’s sexual abuse scale	Y	Y	NR
Deb et al. (2009) [49]	13–18	Sexual Abuse Screening Questionnaire	NR	NR	NR
Deb et al. 2011 a# [50]	13–18	Sexual Abuse Screening Questionnaire	Y	Y	NR
Deb et al. 2011 b# [51]	13–18	Sexual Abuse Screening Questionnaire	Y	Y	NR
Banerjee et al. (2008) [52]	8–14	Not stated	NR	NR	NR
Jangam et al. (2015) [53]	18–50	ISPCAN Child Abuse Screening Tool- Retrospective(ICAST-R)	Y	Y	NR
Reed et al. (2013) [23]	18–40	Not stated	NR	Y	Y
Silverman et al. (2014) [54]	> 18	Not stated	NR	NR	Y
Gaidhane et al. (2008) [55]	11–19	Non- Standardized	NR	NR	NR
Bal et al. (2010) [12]	11–15	Not stated	NR	NR	Y
Tomori et al. 2016** [56]	≥18 years	Not stated	NR	NR	Y
Sahay et al. 2008 [57]	15–26	FGDS and IDIs	NR	NR	NR
Karandikar et al. 2013 [58]	20–60	Semi-structured Interview	NR	NR	Y
Rashid 2012 [24]	13–18	IDS’s	Y	NR	Y
Basu 2012 [59]	Not specified	IDIs, participant observations and journal entries	NR	NR	NR
Karandikar et al. 2013 [28]	20–33	Semi-structured Interview	NR	NR	NR
Magar 2013 ^#^ [14]	12–18	FGDs and IDIs	NA	NA	NA
Mimiaga 2015 [60]	> 18	IDIs, FGDs and KIs	NR	NR	Y
Gupta 2009 [16]	14–30	Case-records Narratives	NR	NR	NR
Sahoo 2015 [15]	16–24	IDI’s	NR	NR	NR
Chakrapani 2008 [61]	21–52	IDI’s	NR	NR	Y
Sinha 2015 [62]	22–50	Participant observation, Life-history interviews, and IDIs	NR	NR	NR
Pillai et al. (2011) ^##^ [63]	16–18	Non-standardized questionnaire	NR	Y	NR
Balaji et al. (2011) ^##^ [64]	16–18	Non-standardized questionnaire	Y	Y	NR

*NR* not reported, *NA* not applicable; *Y* Yes, *N* No

# Data based on an intervention study

## Data based on the same Intervention Study. The study was conducted with respondents between 16 and 24 years of age, but the analysis was restricted to respondents between 16 and 18 years of age for the purpose of this review

**Mixed Methods study

***Ministry of Women and Child Development (MoWCD)

****International Society for the Prevention of Child Sexual Abuse and Neglect Tool-Retrospective (ISPCAN)

### Informed consent

Informed consent is the process by which researchers convey to potential participants and/or their guardians the purpose and activities of a study, its associated risks and benefits, and right to withdrawal. Further, it provides participants an opportunity to ask questions before deciding whether to participate. Domestic and international guidelines recommend obtaining consent from both the parent or guardian and assent from the children in accord with their developmental level and decision-making capacity, in research involving minors [2, 4, 9–11]. In cases where parental consent cannot be obtained due to children being from shelter or runaway homes, street children, or in circumstances when caregivers are themselves perpetrators, guidelines recommend consent be obtained from the local guardians (i.e. non-governmental organization (NGO) staff, police officials). The ICMR guidelines discuss that for research related to child abuse, parental (or legal guardian) consent can be waived but that ethics committee should suggest an alternate mechanism to safeguard the child [10].

In our review, equal frequencies of quantitative (65% or 26/40) and qualitative studies (64% or 7/11) reported obtaining informed consent. Out of these studies, 42% (13/31) studies specified whether the consent was obtained verbally or in writing. As recommendations for obtaining informed consent differ by age, we categorized and then analyzed the studies in our review according to the age ranges of the populations they surveyed: i) participants ≤18 years; ii) participants ≥18 years and iii) participants spanning the age range of 8–49 years.

Among the 25 studies that included participants of age ≤ 18 years, 24% (6/25) obtained consent from both the study participant and parent or local guardian, and 44% (11/25) obtained consent from the study participants only. The latter studies were largely conducted in schools and observational/shelter homes, where prior permission to conduct the study was obtained from the respective institutional authorities. Only one study (of street children) reported taking informed consent solely from a guardian (i.e. police personnel and NGO staff members) [12]. In 20% (5/25) studies, whether informed consent was obtained was not reported, and in 8% (2/25) informed consent was deemed not necessary as the research was limited to data extraction from medical records [13, 14].

Among the 17 studies that included participants in the age ≥ 18 years, 71% (12/17) reported consent was obtained from the study participants, while 29% (5/17) did not report information about consent provision. Of note, all of the studies that did not report obtaining consent were cross-sectional and obtained CSV data from FSWs.

Lastly, seven studies included participants spanning the age range of 8–49 years. Out of the seven, three studies did not report information on obtaining consent, one study reported obtaining consent from both the parent and the participant [15], and one reported obtaining consent from the participant alone [16]. Of note, none of the 25 studies surveying CSV in participants age ≤ 18 years, nor the seven studies spanning age 8–49 years, reported discussing with the participant and/or guardian the limits of confidentiality given the mandatory reporting laws for child abuse in India.

### Confidentiality

Another key principle is maintaining privacy and confidentiality of data to protect participants from potential stigma and reprisal from the perpetrator. Sixty-three percent (63% or 32/51) assured participants about maintaining confidentiality of information. Out of the 32 studies, 24 (75%) reported measures that were used to enhance data confidentiality. Twenty-nine percent (29% or 7/24) reported use of secure environments in addition to data confidentiality. Forty-six percent (46% or 11/24) reported changing the names of the participants or not collecting identifying information in the questionnaires and 3/24 studies reported using a secure environment like conducting interviews in private settings or a comfortable safe place where confidentiality could be maintained. Two (2/24) reported de-identifying study documents (i.e. collecting and editing data the same day or destroying audio files). Nineteen (19/51) studies did not report information on confidentiality. Out of these, 5/19 studies were based on secondary data analysis and 14/19 did not report any information. None of the studies reported using computer-based technologies to collect de-identified data to circumvent mandatory reporting laws. Also, none reported whether or not they contacted legal or law enforcement authorities if ongoing CSV was noted.

### Selection, training, and protection of study team members

Key to the conduct of CSV research is the assurance of trained (i.e. in research ethics, nonjudgmental and respectful communication, facilitating referral to support services, minimizing re-traumatization)and qualified research team members who are involved in the collection of data [4, 9–11]. Additionally, protections should be in place for the research staff to assess for emotional trauma and physical harm *they* may incur as a result of collecting the data, with referral to counseling or other support resources as necessary.

About 69% (35/51) of studies in our search reported that study team members were trained in the collection of CSV data, of which 74% (26/35) discussed specifics of the training received. Only 11% (4/35) of the studies reported the study team received formal training in ethics, with all four providing an online ethics training module [17–19]. Twenty-three (23/51) reported the qualifications of the research staff, with many using junior public health nurses, clinical psychologist or psychiatric social workers, medical officers, and peer researchers to collect data. No studies discussed safeguards they may have used to assess and address the impact of the research on the emotional and/or physical wellbeing on the study team.

### Validity of CSV measurement methods

Existing literature stresses the ethical obligation by investigators to ensure the validity of the tools and procedures they use to measure violence. This includes surveying a range of CSV behaviors, classified by WHO as “non-contact sexual abuse,” “contact sexual abuse,” and “forced sexual intercourse,” [20] using validated tools, and avoidance of loaded terms like ‘rape’, ‘abuse’ and ‘violence’.

Twenty-two percent (22% or 11/51) studies reported surveying all three WHO classifications in their assessment of CSV. An equal number (18% or 9/51) limited their surveillance to two categories of CSV. Almost half (43% or 22/51) either limited their surveillance to only one form of abuse or used a loaded term like “sexual abuse” to assess CSV. The remaining studies (18% or 9/51), a mix of qualitative and quantitative manuscripts, did not specify how they assessed CSV.

Among the 40 studies using quantitative methods, only 16 studies (40%) reported use of standardized tools. The tools used included the ISPCAN Child Abuse Screening Tool (ICSAT)–C and ICSAT- R, the Childhood Trauma Questionnaire, adapted versions of the questionnaire used in the MoWCD study, Finkelhor’s Sexual Abuse Scale, and the Sexual Abuse Screening Tool. Out of the 11 studies that used qualitative methods, the most common tools used to explore CSV experiences were focus-group discussion and in-depth interviews.

### Measures to minimize participant harm

Guidelines on related topics discuss obligation by the researcher to minimize possible distress or harm caused to the study participant that may result from his/her participation in the study. This includes assessment of risk and provision or referral to counseling and medical services as necessary.

Only 25% (13/51) of studies discussed such methods. Among them, 54% (7/13) reported providing counselling services to participants as necessary, 31% (4/13) reported referring participants to psychiatric or general services if they appeared in distress [21–23], one reported specific efforts to not re-traumatize participants [24], and one qualitative study requested participants to participate in a one-on-one in-depth interview of convenience to them if they were apprehensive in participating in the focus group discussion [25].

### Participant compensation

Guidelines warn against incentives or inducements to participate in research, particularly for youth [10]. Among the 51 studies included in the review, half (49% or 25/51) included participants of the age ≤ 18 years. Of these, more than half (19/25) did not report any information regarding compensation or incentives given to participants, two reported giving refreshments as incentive to participate [26, 27], one reported giving monetary compensation (Rupees 100 or USD $2) to participants (street children) for the loss of working hours [12], and 2/25 did not give compensation as the study was limited to secondary data analysis. Among the 51 studies, 17 studies included participants of age ≥ 18 years. About half (8/17) of them did not report any information regarding compensation or incentives. Among the remaining (9 studies) the vast majority (7/9) provided participants with monetary compensation (USD $2–$4), one provided reimbursement for transportation [23], and one reported giving ‘health kits’ to participants (FSW’s) that included soap, hand towels, sanitary napkins, toothpaste, toothbrush and a comb [28]. About 14% (7/51) included populations spanning the age group between 8 and 49 years, none of which reported information on incentives given to the participants.

## Discussion

In recent years, there has been an India-wide multisector movement to develop and implement strategies to effectively prevent and address CSV. This includes government introduction of the Integrated Child Protection Scheme in 2009, the passing of the POCSO Act in 2012, and a rapid rise in research dedicated to examining the epidemiology and development of evidence-based CSV interventions [29]. This review provides a comprehensive summary of the ethical practices reported by the prior decade of India CSV scientific literature in the context of ethical and safety recommendations laid forth by international gender-based violence experts. It was by no means intended to be a critique of existing studies, but rather a synthesis of the literature to help direct the development of future ethical guidelines for the conduct and reporting of CSV research in India. Thus, data summarized here, along with the resulting questions and conclusions, has implications for guideline developers, research ethics committees, funding agencies, investigators, and journal editors, who are ultimately responsible for ensuring study conclusions are valid and published in a safe manner.

First, we begin with a frank discussion of the limitations of the review, which largely stem from the absence of existing CSV guidelines. The gap in CSV-specific recommendations led to our development of the ethics checklist based on international guidelines about related topics (i.e. domestic violence, sexual violence, biomedical research in children), which while relevant, were not designed to protect individuals engaged in CSV research. Further, although the reference guidelines were chosen in consultation with India CSV experts and the checklist was finalized after discussion and consensus by all authors, there remains the potential for bias in item inclusion. Second, we limited our review to the information provided in the manuscripts, rather than contacting authors for additional information. Therefore, this review represents the ethics and safety methods *reported* and may not reflect the entirety of the protocols *followed* by the investigators. Nonetheless, the review highlights the need for journals accepting CSV studies to establish a minimum ethical reporting standard and for research ethics committees to set a minimum criteria regarding ethical procedures to be included in CSV study protocols.

First, we recommend that all manuscripts that include data on CSV be required to report the ethical procedures listed in Table 2, and that journals considering CSV manuscripts ensure that each of the Table 2 points have been adequately addressed. This includes a statement that necessary approvals from institutional review board or ethics committee have been obtained (not found in one-third of reviewed studies), along with informed consent from the participant and permission from the parent or guardian if the participant is a minor (not found in one-fifth of reviewed studies). The informed consent process should include provision of information to the participants in understandable, transparent language about the research methods, the degree of participation expected of them and their caretakers, and the associated risks and benefits so they can estimate the associated burden and determine whether they are prepared to partake. Related guidelines further highlight the need to provide the participant with information during the consent process about the limits of confidentiality due to mandatory reporting laws as well as the right to voluntary withdrawal, but simultaneously raise concern about the minor’s capacity to understand these concepts [4, 10, 11]. While none of the studies in our review reported providing this information, it is possible it was included in the consent documents. We recommend ethics committees verify that this information is included in the consent in lieu of 2012 POCSO regulations. To prevent coercion during the informed consent process, we suggest that ethics committee ensure that compensation provided to participants is minimal, particularly when conducting research with minors. But, we simultaneously recognize that compensation should be tailored to the population (i.e. compensation of lost wages for working street children as was done by Bal et al. 2010) [12].

Second, our review highlighted many methods for optimizing participant confidentiality used in recent studies (i.e. ensuring a secure environment for data collection, not collecting identifying information, replacing names with pseudonyms, or de-identifying stored data, and confidentiality procedures for data storage), but investigators could also adopt additional protective measures used in the global CSV literature (i.e. coaching minors to not share their responses with others for their own protection and using computer technology to maintain anonymity to circumvent mandatory reporting laws). Further, none of the articles discussed purposefully breaching confidentiality to disclose ongoing CSV to legal authorities, perhaps because of the general perception of reporting being unhelpful and potentially harmful to participants. We recommend that either data be collected in an anonymous manner to circumvent mandatory reporting laws or that authors publishing data about ongoing or recent CSV disclose whether it was reported to authorities (as is legally required).

Third, related guidelines stress the need for adequate training of study team members who are involved in the data collection process (as was reported by two-thirds of the studies in our review), that investigators have experience in child socio-behavioral and/or clinical sciences, and that study team members have experience in working with children [10, 11]. We recommend that at minimum the study team receive training in basic research ethics, safety and legal issues associated with CSV research, proper rapport building (critical to ensuring the validity of the data collected), and methods for referring participants with reported or suspected abuse histories to support services. While a few of the studies in our review reported providing ethics training to study team members, extensive training is clearly necessary for research on this sensitive topic. Further, as the research can also negatively impact the emotional wellbeing and physical safety of the staff, we recommend frequent debriefing “check in” meetings with the team with referral to counseling resources as necessary and breaks in prolonged periods of data collection, as is also recommended by the WHO domestic violence guidelines [7].

Next, to address the moral obligation to measure and report CSV estimates in a valid manner (as underestimation could divert much needed national resources to prevent and address CSV), we recommend that studies measuring CSV assess all three WHO classifications of CSV (non-contact sexual abuse, contact sexual abuse, and forced sexual intercourse) and use validated CSV instruments where possible. Additionally, we recommend that all studies collecting primary CSV data be required to report how they assessed participant emotional distress (i.e. emotional trauma resulting from participation) and whether they provided on-site counseling or referral to counseling for participants in distress.

Lastly, the process of research should be based on a partnership between all involved. The ICMR guidelines specifically recommend involvement of a youth advisory committee in the planning phase of adolescent community-based research [10]. We suggest that engagement should include individuals with background characteristics similar to the planned research participants in the planning, development and implementation of the research process from initial stages. Doing so will not only help contribute to the validity and efficacy of the study design, but will also potentially reduce the “object status” experienced by many CSV survivors and provide an opportunity for their empowerment.

## Conclusions

In conclusion, our review of the past decade of India CSV literature in the context of existing international guidelines about other forms of gender-based violence, begins to lay a foundation for the development of culturally-tailored recommendations for the conduct and reporting of CSV research in India and similar settings worldwide.

## Abbreviations

CSV: Child Sexual Violence
MWCD: Ministry of Women and Child Development
NGO: Non-Governmental Organization
POCSO: Protection of Children from Sexual Offenses Act

## References

[1] World Health Organization. Guidelines for Medico-legal Care for Victims of Sexual Violence 2003. http://www.who.int/violence_injury_prevention/resources/publications/en/guidelines_chap7.pdf, http://apps.who.int/iris/bitstream/handle/10665/42788/924154628X.pdf?sequence=1. (accessed 8 Aug 2017).

[2] Ministry of Women and Child Development. Study on Child Abuse: India 2007. 2007. https://www.childlineindia.org.in/pdf/MWCD-Child-Abuse-Report.pdf (accessed 8 Aug 2017).

[3] United Nations Children's Fund. Hidden in Plain Sight: A statistical analysis of violence against children 2014. https://www.unicef.org/publications/index_74865.html. (accessed 8 Aug 2017).

[4] CP MERG. Ethical Principles, Dilemmas, and Risks in Collecting Data on Violence against Children: a review of available literature, Statistics and Monitoring Section/Division of Policy and Strategy, UNICEF, New York. 2012. https://data.unicef.org/wp-content/uploads/2015/12/EPDRCLitReview_193.pdf (accessed.

[5] Arpan. Recounting Abuse, Reporting Abusers: reflections from survivors on mandatory reporting. http://www.arpan.org.in/arpans-research-on-mandatory-reporting-yahoo-com/ (accessed 9 Aug 2017).

[6] Veena AS, Chandra PS (2007). A review of the ethics in research on child abuse. Indian J Med Ethics.

[7] World Health Organization. Putting Women First: Ethical and Safety Recommendations for Research on Domestic Violence Against Women 2001. (accessed.

[8] Jewkes R, Dartnell E, Sikweyiya Y (2012). Ethical and safety recommendations for research on the perpetration of sexual violence. Sexual Violence Research Initiative.

[9] UNICEF Guidelines on the Protection of Child Victims of Trafficking 2006. https://www.unicef.org/eca/0610-Unicef_Victims_Guidelines_en.pdf, https://www.unicef.org/protection/Unicef_Victims_Guidelines_en.pdf. (accessed 10 August 2017).

[10] Rasaily R. National Ethical Guidelines for Biomedical Research Involving Children 2017. (accessed.

[11] World Health Organization. Responding to Children and Adolescents Who Have Been Sexually Abused: WHO Clinical Guidelines 2017. (accessed. 29630189

[12] Bal B, Mitra R, Mallick AH, Chakraborti S, Sarkar K (2010). Nontobacco substance use, sexual abuse, HIV, and sexually transmitted infection among street children in Kolkata, India. Subst Use Misuse.

[13] Silverman JG, Decker MR, Gupta J (2007). Experiences of sex trafficking victims in Mumbai, India. Int J Gynaecol Obstet.

[14] Magar V (2012). Rescue and rehabilitation: a critical analysis of sex Workers’ Antitrafficking responses in India. Signs.

[15] Sahoo KC, Hulland KR, Caruso BA (2015). Sanitation-related psychosocial stress: a grounded theory study of women across the life-course in Odisha, India. Soc Sci Med.

[16] Gupta J, Raj A, Decker MR, Reed E, Silverman JG (2009). HIV vulnerabilities of sex-trafficked Indian women and girls. Int J Gynaecol Obstet.

[17] Zolotor AJ, Runyan DK, Dunne MP (2009). ISPCAN child abuse screening tool Children's version (ICAST-C): instrument development and multi-national pilot testing. Child Abuse Negl.

[18] Bhilwar M, Upadhyay RP, Rajavel S, Singh SK, Vasudevan K, Chinnakali P (2015). Childhood experiences of physical, emotional and sexual abuse among college students in South India. J Trop Pediatr.

[19] Dunne MP, Zolotor AJ, Runyan DK (2009). ISPCAN child abuse screening tools retrospective version (ICAST-R): Delphi study and field testing in seven countries. Child Abuse Negl.

[20] Andrews G, Corry J, Slade T, Issakidis C, Swanston H, Ezzati M, Lopez AD, Rodgers A, Murray CJL (2004). Child sexual abuse. Comparative quantification of health risks: global and regional burden of disease attributable to selected major risk factors.

[21] Shahmanesh M, Wayal S, Cowan F, Mabey D, Copas A, Patel V (2009). Suicidal behavior among female sex workers in Goa, India: the silent epidemic. Am J Public Health.

[22] Shahmanesh M, Cowan F, Wayal S, Copas A, Patel V, Mabey D (2009). The burden and determinants of HIV and sexually transmitted infections in a population-based sample of female sex workers in Goa, India. Sex Transm Infect.

[23] Reed E, Silverman JG, Stein B (2013). Motherhood and HIV risk among female sex workers in Andhra Pradesh, India: the need to consider women's life contexts. AIDS Behav.

[24] Rashid J (2012). An analysis of self-accounts of children-in-conflict-with-law in Kashmir concerning the impact of torture and detention on their lives. Int Soc Work.

[25] Sahay S (2013). Making of victim a patient: sexually abused children and the consequences of unprofessional help. Psychol Stud.

[26] Charak R, Koot HM (2014). Abuse and neglect in adolescents of Jammu, India: the role of gender, family structure, and parental education. J Anxiety Disord.

[27] Charak R, Koot HM (2015). Severity of maltreatment and personality pathology in adolescents of Jammu, India: a latent class approach. Child Abuse Negl.

[28] Karandikar S, Gezinski LB, Meshelemiah JCA (2013). A qualitative examination of women involved in prostitution in Mumbai, India: the role of family and acquaintances. Int Soc Work.

[29] Choudhry V, Dayal R, Pillai D, Kalokhe AS, Beier K, Patel VH. Child sexual abuse in India: a systematic review. Pending review at PLOS ONE August 2017. 10.1371/journal.pone.0205086 PMC6177170 30300379

[30] Jaisoorya TS, Janardhan Reddy YC, Thennarasu K, Beena KV, Beena M, Jose DC (2015). An epidemological study of obsessive compulsive disorder in adolescents from India. Compr Psychiatry.

[31] Sahay S, Nirmalkar A, Sane S, Verma A, Reddy S, Mehendale S (2013). Correlates of sex initiation among school going adolescents in Pune, India. Indian J Pediatr.

[32] Miller E, Das M, Tancredi DJ (2014). Evaluation of a gender-based violence prevention program for student athletes in Mumbai, India. J Interpers Violence.

[33] Hasnain N, Kumar D (2006). Psychological well-being of women reporting sexual abuse in childhood. J Indian Acad Appl Psychol.

[34] Das M, Ghosh S, Verma R (2014). Gender attitudes and violence among urban adolescent boys in India. Int J Adolesc Youth.

[35] Deb S, Walsh K (2012). Impact of physical, psychological, and sexual violence on social adjustment of school children in India. Sch Psychol Int.

[36] Deb S, Modak S (2010). Prevalence of violence against children in families in Tripura and its relationship with socio-economic factors. J Inj Violence Res.

[37] Krishnakumar P, Satheesan K, Geeta M, Sureshkumar K (2014). Prevalence and spectrum of sexual abuse among adolescents in Kerala, South India. Indian J Pediatr.

[38] Jaya J, Hindin MJ (2007). Nonconsensual sexual experiences of adolescents in urban India. J Adolesc Health.

[39] Nayak MB, Korcha RA, Benegal V (2010). Alcohol use, mental health, and HIV-related risk behaviors among adult men in Karnataka. AIDS Behav.

[40] Sahay S (2010). Compelled subjugation and forced silence: sexually abused girls and their family members: a case study of Western Madhya Pradesh (India). Int J Adolesc Youth.

[41] Pillai A, Patel V, Cardozo P, Goodman R, Weiss HA, Andrew G (2008). Non-traditional lifestyles and prevalence of mental disorders in adolescents in Goa, India. Br J Psychiatry.

[42] Bhattacharyya SK, Saha SP, Pal R (2012). Rape among women and girls presenting at a gynecological emergency department, North Bengal Medical College, Darjeeling, India. Int J Gynaecol Obstet.

[43] Kar N, Koola MM (2007). A pilot survey of sexual functioning and preferences in a sample of English-speaking adults from a small south Indian town. J Sex Med.

[44] Silverman JG, Decker MR, Gupta J, Maheshwari A, Patel V, Raj A (2006). HIV prevalence and predictors among rescued sex-trafficked women and girls in Mumbai, India. J Acquir Immune Defic Syndr.

[45] Silverman JG, Raj A, Cheng DM (2011). Sex trafficking and initiation-related violence, alcohol use, and HIV risk among HIV-infected female sex workers in Mumbai, India. J Infect Dis.

[46] Deb S (2008). Mental disposition of commercial sex workers (CSWs) with HIV/AIDS. J Indian Acad Applied Psychology.

[47] Devine A, Bowen K, Dzuvichu B, Rungsung R, Kermode M (2010). Pathways to sex-work in Nagaland, India: implications for HIV prevention and community mobilisation. AIDS Care.

[48] Bhat DP, Singh M, Meena GS (2012). Screening for abuse and mental health problems among illiterate runaway adolescents in an Indian metropolis. Arch Dis Child.

[49] Deb S, Mukherjee A (2009). Impact of sexual abuse on personality disposition of girl children. J Indian Acad Applied Psychology.

[50] Deb S, Mukherjee A (2011). Background and adjustment capacity of sexually abused girls and their perceptions of intervention. Child Abuse Rev.

[51] Deb S, Mukherjee A, Mathews B (2011). Aggression in sexually abused trafficked girls and efficacy of intervention. J Interpers Violence.

[52] Banerjee SR, Bharati P, Vasulu TS, Chakrabarty S, Banerjee P (2008). Whole time domestic child labor in metropolitan city of Kolkata. Indian Pediatr.

[53] Jangam K, Muralidharan K, Tansa KA, Aravind Raj E, Bhowmick P (2015). Incidence of childhood abuse among women with psychiatric disorders compared with healthy women: data from a tertiary care Centre in India. Child Abuse Negl.

[54] Silverman JG, Saggurti N, Cheng DM (2014). Associations of sex trafficking history with recent sexual risk among HIV-infected FSWs in India. AIDS Behav.

[55] Gaidhane AM, Syed Zahiruddin Q, Waghmare L, Shanbhag S, Zodpey S, Joharapurkar SR (2008). Substance abuse among street children in Mumbai. Vulnerable Children and Youth Studies.

[56] Tomori C, McFall AM, Srikrishnan AK (2016). The prevalence and impact of childhood sexual abuse on HIV-risk behaviors among men who have sex with men (MSM) in India. BMC Public Health.

[57] Sahay S (2008). Socio-cultural factors and young sexual offenders: a case study of Western Madhya Pradesh (India). Int J Adolesc Youth.

[58] Karandikar S, Gezinski LB (2013). Intimate partner violence and HIV risks among female sex Workers of Mumbai, India. J Ethn Cult Divers Soc Work.

[59] Basu A (2010). Communicating health as an impossibility: sex work, HIV/AIDS, and the dance of Hope and hopelessness. Southern Commun J.

[60] Mimiaga MJ, Closson EF, Thomas B (2015). Garnering an in-depth understanding of men who have sex with men in Chennai, India: a qualitative analysis of sexual minority status and psychological distress. Arch Sex Behav.

[61] Chakrapani V, Newman PA, Shunmugam M (2008). Secondary HIV prevention among kothi-identified MSM in Chennai, India. Cult Health Sex.

[62] Sinha S (2015). Reasons for Women's entry into sex work: a case study of Kolkata, India. Sexu Cult.

[63] Pillai A, Andrews T, Patel V (2009). Violence, psychological distress and the risk of suicidal behaviour in young people in India. Int J Epidemiol.

[64] Balaji M, Andrews T, Andrew G, Patel V (2011). The acceptability, feasibility, and effectiveness of a population-based intervention to promote youth health: an exploratory study in Goa, India. J Adolesc Health.

